# Possible Modulation of Vascular Function Measures in Rheumatoid Arthritis by Homocysteine

**DOI:** 10.1155/2018/8498651

**Published:** 2018-07-02

**Authors:** Mahmoud A. Alomari, Omar F. Khabour, Khaldoon Alawneh, Rania A. Shammaa

**Affiliations:** ^1^Division of Physical Therapy, Department of Rehabilitation Sciences, Jordan University of Science and Technology, Irbid, Jordan; ^2^Department of Medical Laboratory Sciences, Jordan University of Science and Technology, Jordan; ^3^Department of Internal Medicine, Faculty of Medicine, Jordan University of Science and Technology, Irbid, Jordan; ^4^Division of Rheumatology, Department of Medicine, King Abdulla Hospital, Irbid, Jordan; ^5^Department of Applied Biology, Faculty of Art and Science, Jordan University of Science and Technology, Irbid, Jordan

## Abstract

The effect of homocysteine on cardiovascular diseases is still equivocal, especially in rheumatoid arthritis patients. In this investigation, the association between homocysteine with blood flow and vascular resistance in rheumatoid arthritis was examined. Serum levels of homocysteine were determined in thirty-one rheumatoid arthritis patients and nineteen apparently healthy subjects using ELISA. Additionally, strain-gauge plethysmography was used to determine both forearm blood flow and vascular function at rest and after occlusion. Forearm occlusion blood flow (patients: 21.9 ± 6.55 versus control: 25.5 ± 6.10ml/100mL/min) was lower (p < 0.05) while occlusion vascular resistance (patients: 4.77 ± 2.08 versus controls 3.05 ± 0.96U) was greater (p < 0.01) in rheumatoid arthritis than in the controls. Level of serum homocysteine was similar (p = 0.803) in rheumatoid arthritis group and healthy group. In addition, level of serum homocysteine was correlated with resting blood flow (r = −0.41; p < 0.02) and resting vascular resistance (r = 0.31, p < 0.05) in the patients group. The study confirms altered vascular function in rheumatoid arthritis. Uniquely, the results show that homocysteine was related to resting, but not postischemia, vascular measures. These relationships indicate that homocysteine might impact the vasculature in rheumatoid arthritis.

## 1. Introduction

Rheumatoid arthritis is an autoimmune disease associated with access proinflammatory cytokines that target the joint synovium resulting in tender, warm, swollen, and stiff joints [1]. Subsequently, the patients suffer from limited range of movements, as well as diminished functionality, performance of activities of daily living, and eventually quality of life [1]. However, since these cytokines seep into the circulation [1], hyperinflammatory and hyperautoimmune responses affect the cardiovascular (CV) system [2, 3]. The vasculature, in particular, undergoes a variety of changes resulting in greater risk for developing CV diseases (CVDs) [4]. In fact, the risk of CVDs is comparable to those with type 2 diabetes and is a main factor of excess mortality in rheumatoid arthritis [2, 3].

Traditional risk factors play a key role in the development atherogenesis in rheumatoid arthritis [5], However, 15–20% of the patients are with “normal” traditional CV risk profile [3] and thus might not qualify for prevention interventions [6]. Scientists, therefore, have tried to identify other risk factors to help predicting portion of CVDs in rheumatoid arthritis [7, 8].

Homocysteine has been implicated in atherogenesis of coronary, cerebral, and peripheral circulations [9]. High homocysteine level can predict CV-related events, including death, in variety of diseases [10–14]. Additionally, homocysteine has been shown to independently double the risk of CVDs with every 5 *μ*mol/L increase [12], while higher homocysteine levels lower survival rate [15].

Despite the accumulating evidence describing the effect of homocysteine on CV function, few studies with conflicting results have examined CV effects of homocysteine in rheumatoid arthritis. Studies have shown that homocysteine is related to carotid structure [16] and might, at least partially, explain risk of developing CVDs [7]. Conversely, homocysteine was not related to L-arginine (Arg) [17], while it was related to asymmetric dimethylarginine in rheumatoid arthritis, essential for nitric oxide (NO) production and vascular function. Studies that examined the impact of increased homocysteine concentration on vascular function in rheumatoid arthritis are needed. Therefore, the current study will determine circulatory homocysteine levels and vascular function in rheumatoid arthritis. Subsequently the associations between homocysteine levels with vascular function indices will be examined.

## 2. Materials and Methods

### 2.1. Recruitment and Design

Rheumatoid arthritis participants were recruited from the outpatient clinics of King Abdullah University Hospital in the northern part of Jordan. All patients met the 1987 ACR (American College of Rheumatology) criteria [18] and were diagnosed by a rheumatology consultant. Apparently healthy individuals were recruited from the local community to serve as controls. Individuals diagnosed with hypertension, smoking, unstable myocardial ischemia, angina, diabetes mellitus, anemia, chronic lung diseases, hypercholesterolemia, renal failure, severely obese, atherosclerosis, and major CV risk factors were excluded from the study. Subjects with these conditions were excluded because previous literature has shown strong impact for these heath complications on cardiovascular measures. All patients continued taking prescribed medications.

The objectives and procedures were orally and in written form explained to all participants before they signed the consent form, which was approved by the Institutional Research Board. Subsequently, plasma serum homocysteine, glucose, total lipid profile concentration, ESR, forearm blood flow, and vascular resistance were determined.

### 2.2. Evaluation of Serum Homocysteine

Blood samples were drawn, from each patient after 12–14 hours' fasting, by venipuncture into 5-mL plain evacuated serum tubes and then placed in ice prior to analyzing. The red blood cells were separated from serum by centrifugation and transfer to an AxYAM sample cup. The remaining serum was then transferred to a clean container and stored frozen at −20 degree for future analysis. Serum levels of homocysteine were determined on the same day for all participants using an Abbott diagnostics kit (Abbott Park, 1L 60064, USA). Briefly, reduction of bound homocysteine was achieved by the addition of dithiothreitol to produce S-adenosyl-1-homocysteine. This product competes with fluorescein labeled–S-adenosyl-cysteine supplied by the kit for binding to a specific monoclonal antibody. The polarized fluorescent light was then measured and its intensity is inversely proportional to homocysteine concentration in the specimen. Serum homocysteine concentrations were calculated by the Abbott AxYAM. According to the kit, the standard curve is linear with homocysteine levels values between 2 and 50*μ*mol/L.

### 2.3. Blood Glucose, Lipid Profile, and ESR Measurements

Glucose, lipid profile (HDL, LDL, and cholesterol), and ESR were analyzed at the biochemistry center at the hospital. Biosafety measures were followed according to the hospital regulations and procedures.

### 2.4. Vascular Function Measurements

Strain-gauge plethysmography was used to examine forearm vascular function. The technique is noninvasive based on changes in limb blood flow rate following pressure manipulations of pneumatic cuffs placed on the upper and distal arm [19]. Resting and postocclusion blood flow were measured via a mercury in silastic strain gauge [20]. In brief, upon arrival, the participant assumed supine position for 15 minutes and 2 pneumatic cuffs were wrapped around the upper arm and wrist. Subsequently, resting blood flow was measured as previously described [20]. Following 5–10 minutes, the upper arm cuff was inflated to suprasystolic blood pressure (BP) for 5 minutes. Thereafter, occlusion blood flow was recorded at 25 millimeters/second paper speed and measured after releasing the upper cuff pressure [21]. Immediately before obtaining resting and occlusion blood flow indices, the circulation of the arm was occluded for 60 seconds by filling the wrist cuff to 250 mmHg. Concurrently, heartbeat per minute and BP measurements were acquired from the counter hand at rest and after 5 min of circulation occlusion using standardized procedures.

Forearm resting blood flow was estimated from the slope drawn at the first 2-3 beats of the plethysmographic volume graph generated immediately after venous occlusion. Forearm occlusion blood flow was determined with the best-fit slope drawn at the first 2-3 beats of the forearm volume graph after upper cuff release and calculated as previously described [20]. Forearm resting and occlusion blood flow were expressed as ml of blood/100 ml of tissue/min (ml/100ml/min). Resting and postocclusion vascular resistance were calculated as mean arterial pressure/forearm BF and expressed as unit (U).

### 2.5. Statistical Analysis

All statistical analysis was performed using SPSS (version 17, Chicago, IL). Primary and secondary study indices in rheumatoid arthritis versus healthy individuals were analyzed using independent *t*-tests and presented as mean ± SD. Additionally, Pearson's product correlation tests were used to examine the associations between serum homocysteine concentrations and vascular function measures and presented as r-values and p values. The power for testing correlations between vascular indices and homocysteine levels was 68. This was obtained using online statistical software: http://powerandsamplesize.com/Calculators/. For all tests, *α* was 0.05. With respect to *β*, it was less than 0.32. The Shapiro-Wilk (S-W) test was used to examine the normal distribution for age, height, and weight, as they are often not normally distributed. A p value was set at 0.05.

## 3. Results

### 3.1. Participant Characteristics

Thirty-one rheumatoid arthritis patients and 19 controls participated in the study. As shown in Table 1, gender distribution, height, weight, age, body mass index, and body fat percentage (BF%) were not different while waist/hip ratio was different in the rheumatoid arthritis and control group. In addition, CV hemodynamics were different (p<0.05) between the two groups, while blood glucose, total cholesterol, LDL, and HDL, were similar between both groups. The mean disease activity score (DAS28) for RA patients was 4.85±1.3. The data in Table 1 show that the patients mean disease duration was ~3 years. A total of 16 patients (51.6%) were taking methotrexate, among which 2 (6.4%) were also taking Adalimumab. All patients were taking folate while no other DMARDs or NSAIDs were prescribed.

### 3.2. Test of Normality

The W-S p values for age, weight, and height among the patients were 0.918, 0.070, and 0.177, respectively.

### 3.3. Effect of Rheumatoid Arthritis on Homocysteine Levels

No significant differences (p = 0.8) were found in homocysteine levels between the rheumatoid arthritis patients (*μ* = 10.6 ± 3.03) and the controls (*μ* = 10.4 ± 2.21). Blood homocysteine level was also not different (p = 0.3) between the rheumatoid arthritis patients with (*μ* = 11.1 ± 3.49) and without (*μ* = 10.1 ± 2.34) methotrexate (MTX) and no correlations (r = −0.5; p = 0.1) were found between MTX dose and homocysteine levels.

### 3.4. Effect of Rheumatoid Arthritis on Vascular Function Indices

Table 2 presents vascular function indices at rest and after occlusion for the patients and the controls. There were no differences in forearm resting blood flow and resting vascular resistance in the patients versus controls, whereas occlusion blood flow was lower (p < 0.05) and occlusion vascular resistance was higher (p < 0.01) in the rheumatoid arthritis patients group.

### 3.5. Relationships between Total Homocysteine Levels and Vascular Measures

**Figure 1:**
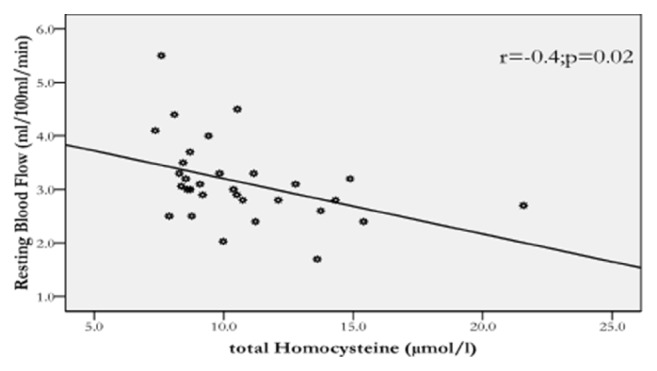
The relationship of total homocysteine level with resting blood flow in the patients (*r* = −0.4; *p* = 0.02).

**Figure 2:**
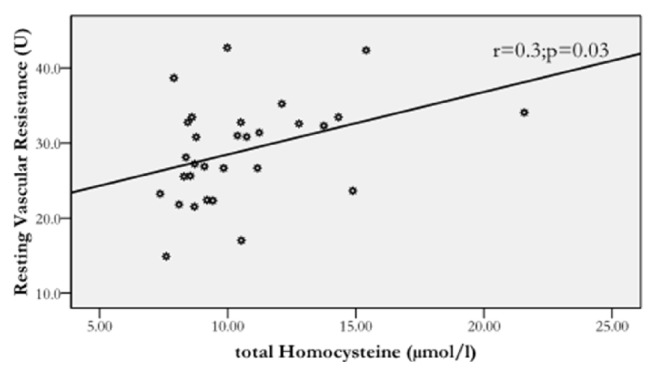
The relationship of homocysteine level with resting vascular resistance in the patients (*r* = −0.3; *p* = 0.03).

Figures 1 and 2 show that homocysteine levels correlated negatively with resting blood flow (r = −0.401, p < 0.05) and positively with resting vascular function (r = 0.31, p < 0.05) in the patients. However, no significant association were detected between serum homocysteine, occlusion blood flow (r = 0.12, p > 0.05), and occlusion vascular resistance (r = −0.103, p > 0.05) in the patients. Furthermore, waist/hip ratio was not related to serum homocysteine level (r = 0.18; p > 0.33) or vascular measures (r^range^ = −0.14/0.26; p^range^ = 0.14/0.8) in the patients. Similarly, BMI was not related to homocysteine (r = 0.08; p = 0.7) or vascular measures (r^range^ = −0.2/0.3; p^range^ = 0.07/0.7) in the patients. Likewise, ESR was not related to serum homocysteine level (r = 0.04; p = 0.81) or vascular measures (r^range^ = −0.002/0.16; p^range^ = 0.4/0.99) among the patients. Finally, no significant associations (p > 0.05) were detected between serum homocysteine and with any of the vascular measures in the control group.

## 4. Discussion

The study was aimed at examining vascular function and serum homocysteine in rheumatoid arthritis participants and healthy controls. Vascular function was diminished whereas homocysteine level was the same in rheumatoid arthritis patients versus the controls. Furthermore, homocysteine levels were related to vascular function in the rheumatoid arthritis patients but not in the controls.

Few studies, with equivocal results, examined homocysteine levels in rheumatoid arthritis patients [7, 22–24]. Data have shown substantial elevation in homocysteine with rheumatoid arthritis due to abnormal homocysteine metabolism [7, 22, 24]. One study have found that homocysteine was 33% greater than healthy counterparts [22]. These abnormalities were attributed to low vitamin B_6_ levels [7] and alteration in either the transsulfuration pathway or the regulation of the homocysteine cycle [24]. Conversely, similar to the current study, Van Doornum showed no differences in homocysteine levels between the patients and healthy participants. The authors postulated that normal B vitamin levels prevented abnormal changes in the homocysteine pathway [25]. All the patients in the current study were on B vitamin supplements. Therefore, given the importance of B vitamins for homocysteine metabolism, we think that adequate B vitamins might have promoted the conversion of homocysteine to cysteine or methionine [26, 27] and thereby prevented homocysteine elevation in the patients of the current study.

The comparisons between the rheumatoid arthritis patients with and without MTX showed no differences in homocysteine levels. Additionally, no correlations (r = −0.5; p = 0.1) were found between MTX dose and homocysteine levels. Consistent with previous studies, these results might indicate that MTX does not affect homocysteine levels [7, 24], especially with sufficient folate supplement [28]. However, a previous report showed increases in homocysteine levels in rheumatoid arthritis patients following MTX treatment [29].

Several “precautionary” steps were taken to avoid the possible effect of external factors on homocysteine metabolism. For example, blood homocysteine levels were evaluated after 12–14 hours' fasting except for the prescribed medications. Additionally, as in Tables 1 and 2 and per the study exclusion criteria, the patients were free from renal failure, hypothyroidism, and diabetes as well as other CVD risk factors, including smoking.

The reduced occlusion blood pressure and increased occlusion vascular resistance in the patients are consistent with previous studies [30–33]. Arosio et al., for example, reported a -29% in peak blood flow (p = 0.05) in rheumatoid arthritis [31]. Similarly, another study showed lower flow mediated dilation in rheumatoid arthritis patients [32]. The blood flow responses to SNP and Ach were also lower in rheumatoid arthritis patients compared with the controls by 30 and 50%, respectively [33]. However, one study showed that homocysteine was not related to Arg, pivotal for NO metabolism and arterial function, despite the introduction of omega-3 fatty acids, vitamin E, vitamin A, copper, and selenium supplementation [17].

Previous studies have attributed these malfunctions to structural and functional changes in the arterial properties. These changes include reduced endothelial function [32] and arterial diameter, responsiveness [34], and elasticity [31, 35] as well as increased intima-media thickness [3]. Bergholm et al. reported that diminished NO bioavailability and/or responsiveness of smooth muscle to endogenous and exogenous NO considered as one of the factors affecting vascular responses to endothelium-dependent and endothelium-independent stimulus [33]. The majority of these arterial modifications have been attributed to hyperinflammatory and hyperimmunoresponses [1, 36, 37].

Figures 1 and 2 show relationships of homocysteine with forearm resting blood flow and resting vascular resistance in the patients. This is the first study to show these relationships suggesting that vascular function might be influenced by homocysteine levels in rheumatoid arthritis patients. In particular, these relationships were not found, in the current and previous studies [38], among healthy individuals, indicating that rheumatoid arthritis might have “augmented” the negative effect of blood homocysteine on vascular function. It is also important to note that these relationships were found despite similar glucose and lipid profile in the patients versus the control. However, given vascular dysfunction is multifactorial, other factors might have contributed to this vascular changes in the current study. Therefore, other more investigations are needed to examine the contribution of other factors, in addition to homocysteine, to vascular dysfunction in rheumatoid arthritis.

We are unaware of previous studies examining these relationships in rheumatoid arthritis, and thus we cannot compare the results. However, previous studies have shown relationships of homocysteine with CVD in type 2 diabetes mellitus [10, 39, 40], renal failure [41], peripheral arterial disease [11], venues thromboembolism [14], and systemic lupus erythematosus [13]. In fact studies have suggested considering homocysteine a predictor for CVDs and death in some diseases (i.e., type 2 diabetes) [40]. Given the current design, we think homocysteine can be used as a biomarker for vascular function in rheumatoid arthritis. Therefore, future longitudinal investigations are certainly demanded to study the exact contribution of homocysteine to CVDs in rheumatoid arthritis.

The exact possible pathophysiological mechanisms for this increase in CV risk are not well-known. Nonetheless, elevated homocysteine is associated with endothelial and arterial structural and functional changes [42]. These changes have been attributed to accelerated oxidative stress [43], inflammation [44], and reduced nitric oxide bioavailability [42], which can promote vascular inflammation and thrombogenesis [42]. In fact, a recent study found that elevated homocysteine in rheumatoid arthritis was related to inflammatory factors [22]. Furthermore, homocysteine increases blood clotting rate by changing the coagulation factor levels, thereby increasing small artery rigidity and vulnerability for plaque and clot formations [27]. These alterations in rheumatoid arthritis are established factors contributing to the development of CVDs [45]. However, future studies are needed to examine these relationship while covariating for inflammatory factors.

The results of the current study show that homocysteine levels did not correlate with occlusion blood flow and occlusion vascular resistance. These results might suggest that ischemia, during upper arm arterial occlusion, abolished the effect of homocysteine on vascular function. Increased shear stress, after the removal of the occlusion cuff, might have stimulated the release of metabolic and endothelium vasodilators and subsequently overcame the harmful effect of homocysteine on the vasculature. These vasodilators, including bradykinin, adenosine, serotonin, hypoxia, VEGF, NO, and prostacyclin, are known for their propitious effect on the vasculature [46].

Improving muscular function might be pivotal in protecting the vascular function in rheumatoid arthritis patients [30, 47, 48]. It is documented that hyperinflammatory status and reduced physical activities are the main causes for diminished muscle size, structure, and function in rheumatoid arthritis [49]. These muscle alterations might have resulted in decreased muscle capacity to release metabolic vasodilators and thus diminished vascular function [49]. Accumulation of waste products, including metabolic vasodilators, during occlusion-induced ischemia might have resulted in augmented vasodilatory response thus disassociation between homocysteine and the vasculature, as observed during resting.

It is postulated that strategies aimed at decreasing inflammation and enhancing muscle size should be implemented. Regular participation in exercise seems an appropriate strategy that can fulfill both aims [47, 50, 51]. Several previous studies have reported that exercise is associated with reduced inflammatory response [52] and increased muscle size and function [53] in rheumatoid arthritis. In fact, previous data have indicated that greater muscular strength was associated with improved vascular function in rheumatoid arthritis [30] and the elderly [47]. However, these are mere speculations in need for further verifications in future mechanistic studies.

Vascular function is diminished and is associated with increased risk of developing CVDs in rheumatoid arthritis. According to the current study, the damaging effect of homocysteine on the vasculature seems to be augmented in rheumatoid arthritis patients. Since systemic hyperinflammatory status is the obvious characteristic of rheumatoid arthritis, these results suggest that inflammation might have exaggerated the effect of homocysteine on the vasculature. This seemingly homocysteine-induced alterations in vascular function was abolished with occlusion-induced ischemia among rheumatoid arthritis patients. Ischemia might have augmented the release of vasodilatory factors, which might have decreased vascular resistance and increased blood flow. Therefore, strategies aimed at improving vascular function and decreasing homocysteine and inflammation might be essential in this population. However, research is indeed needed to further verify these mere speculations.

The main limitation in the study was the small sample size, which might cause a type one error and limited the generalizability of the results. Additionally, small sample size might have prevented us from examining the role of other factors that might have contributed to the relationship of homocysteine with vascular function, factors like gender, age, obesity, blood glucose/lipid profile, inflammation, and physical activity. However, the small sample size was due to difficulties finding rheumatoid arthritis patients without CVDs and/or metabolic disorders. Additionally, the inherited limitations of a cross-sectional design, including that these relationships are not cause-effect, should also be acknowledged. Smoking is a risk factor for CVDs and prevalent among rheumatoid arthritis patients. Therefore, future studies should examine the effect of smoking and other comorbidities on the relationship of homocysteine with vascular function in rheumatoid arthritis. Given inflammation is major concern in rheumatoid arthritis, C reactive protein relationship with CV function should have been examined. In the current study, vascular function was measured with strain-gauge plethysmography, which measures the whole limb blood flow. Future studies should also examine vascular function in a single artery. For example, flow mediated dilation can give a different view of the dynamics of one artery, which can be insightful. Therefore, future studies should be using flow mediated dilation to measure the relationship of homocysteine with vascular function.

## 5. Conclusion

Vascular function was diminished whereas homocysteine levels were similar in the patients versus the control. The unique finding of the study is the relationships of homocysteine with resting blood flow and resting vascular resistance in rheumatoid arthritis. This might suggest that vascular function might be influenced by homocysteine in rheumatoid arthritis patients. The hyperinflammatory status in rheumatoid arthritis might have augmented the effect of homocysteine on the vasculature. However this relationship disappeared with postocclusion vascular measures suggesting that vasodilators, associated with ischemia and abolished the damaging effect of homocysteine on the vasculature. Therefore, we think strategies such as regular participation in exercise aimed at increasing muscle capacity to release metabolic vasodilators might be useful to improve vascular function in these patients.

## Figures and Tables

**Table 1:** Participant characteristics, cardiovascular hemodynamics, and blood lipid parameters.

**Variables**	**Patients (n = 31)**	**Control (n = 19)**	***p *value**
**Demographics**			

Females, n (%)	31 (100)	19 (100)	1.000
Disease duration, months	34.7 ± 42.6		
Age, years	39.3 ± 12.1	37.6 ± 13.7	0.600
Height, cm	161 ± 8.28	159 ± 7.14	0.200
Weight, kg	71.7 ± 17.0	64.6 ± 13.5	0.100
Body mass index, kg/m2	27.2 ± 6.11	25.6 ± .82	0.300
Body Fat, %	31.6 ± 7.71	30.1 ± 6.98	0.400
Waist/hip	0.82 ± 0.15	0.72 ± 0.09	0.005

**Cardiovascular Hemodynamics**			

Heart rate, b/min	78.5 ± 11.5	69.5 ± 8.03	0.001
Systolic pressure, mmHg	119 ± 12.8	108 ± 11.9	0.003
Diastolic pressure, mmHg	73.9 ± 9.74	68.1 ± 8.20	0.010
Mean pressure, mmHg	88.9 ± 10.1	81.4 ± 8.64	0.004
Pulse pressure, mmHg	44.5 ± 8.20	39.8 ± 8.86	0.070

**Blood Lipid Parameters**			

Cholesterol, mmol/l	5.01 ± 1.05	4.61 ± 1.24	0.200
HDL, mmol/l	1.32 ± 0.32	1.37 ± 0.34	0.500
LDL, mmol/l	3.26 ± 0.76	3.05 ± 1.22	0.600
Glucose, mmol/l	4.99 ± 0.76	4.74 ± 0.47	0.100
ESR, Units	63.5 ± 25.7	21.6 ± 9.07	0.001
RF, IU/mL	112.0 ± 127.0		

Data presented as mean ± SD, ESR: erythrocyte sedimentation rate, HDL: high density lipoproteins, and LDL: low density lipoproteins.

**Table 2:** Vascular function indices.

	**Patients (n = 31)**	**Control (n = 19)**	**p value**
**Blood flow, ml/100ml tissue/min**			

Resting	3.11 ± 0.76	3.18 ± 0.74	0.700
Post-occlusion	21.9 ± 6.55	25.5 ± 6.10	0.050

**Vascular resistance, U**			

Resting	29.4 ± 7.32	26.7 ± 5.83	0.100
Post-occlusion	4.77 ± 2.08	3.05 ± 0.96	0.006

Data presented as mean ± SD.

## Data Availability

The data used to support the findings of this study are available from the corresponding author upon request.
